# Serological Evidence of Ebola Virus Infection in Indonesian Orangutans

**DOI:** 10.1371/journal.pone.0040740

**Published:** 2012-07-18

**Authors:** Chairul A. Nidom, Eri Nakayama, Reviany V. Nidom, Mohamad Y. Alamudi, Syafril Daulay, Indi N. L. P. Dharmayanti, Yoes P. Dachlan, Mohamad Amin, Manabu Igarashi, Hiroko Miyamoto, Reiko Yoshida, Ayato Takada

**Affiliations:** 1 Avian Influenza-zoonosis Research Center, Airlangga University, Surabaya, Indonesia; 2 Faculty of Veterinary Medicine, Airlangga University, Surabaya, Indonesia; 3 Institute of Tropical Disease, Airlangga University, Surabaya, Indonesia; 4 Division of Global Epidemiology, Hokkaido University Research Center for Zoonosis Control, Sapporo, Japan; 5 Center for Diagnostic Standard of Agriculture Quarantine, Ministry of Agriculture, Jakarta, Indonesia; 6 Indonesian Research Center for Veterinary Science, Ministry of Agriculture, Bogor, Indonesia; 7 Tropical Disease Hospital, Airlangga University, Surabaya, Indonesia; 8 Division of Bioinformatics, Hokkaido University Research Center for Zoonosis Control, Sapporo, Japan; Tulane School of Public Health and Tropical Medicine, United States of America

## Abstract

Ebola virus (EBOV) and Marburg virus (MARV) belong to the family *Filoviridae* and cause severe hemorrhagic fever in humans and nonhuman primates. Despite the discovery of EBOV (Reston virus) in nonhuman primates and domestic pigs in the Philippines and the serological evidence for its infection of humans and fruit bats, information on the reservoirs and potential amplifying hosts for filoviruses in Asia is lacking. In this study, serum samples collected from 353 healthy Bornean orangutans (*Pongo pygmaeus*) in Kalimantan Island, Indonesia, during the period from December 2005 to December 2006 were screened for filovirus-specific IgG antibodies using a highly sensitive enzyme-linked immunosorbent assay (ELISA) with recombinant viral surface glycoprotein (GP) antigens derived from multiple species of filoviruses (5 EBOV and 1 MARV species). Here we show that 18.4% (65/353) and 1.7% (6/353) of the samples were seropositive for EBOV and MARV, respectively, with little cross-reactivity among EBOV and MARV antigens. In these positive samples, IgG antibodies to viral internal proteins were also detected by immunoblotting. Interestingly, while the specificity for Reston virus, which has been recognized as an Asian filovirus, was the highest in only 1.4% (5/353) of the serum samples, the majority of EBOV-positive sera showed specificity to Zaire, Sudan, Cote d’Ivoire, or Bundibugyo viruses, all of which have been found so far only in Africa. These results suggest the existence of multiple species of filoviruses or unknown filovirus-related viruses in Indonesia, some of which are serologically similar to African EBOVs, and transmission of the viruses from yet unidentified reservoir hosts into the orangutan populations. Our findings point to the need for risk assessment and continued surveillance of filovirus infection of human and nonhuman primates, as well as wild and domestic animals, in Asia.

## Introduction

Ebola virus (EBOV) and Marburg virus (MARV) are enveloped negative-strand RNA viruses belonging to the family *Filoviridae*. While MARV consists of a single species, *Lake Victoria marburgvirus*, five distinct EBOV species are known: *Zaire ebolavirus* (ZEBOV), *Sudan ebolavirus* (SEBOV), *Côte d’Ivoire ebolavirus* (CIEBOV), *Bundibugyo ebolavirus* (BEBOV), and *Reston ebolavirus* (REBOV) [1], [2]. Outbreaks of Ebola and Marburg hemorrhagic fever in humans and nonhuman primates (other than imported cases) have occurred sporadically in central and west Africa, but REBOV was first reported in 1989–1990 by several quarantine facilities in the United States, when wild-caught monkeys (*Macaca fascicularis*) imported from the Philippines became ill, with some dying [1]. In 2008–2009, REBOV infection was occasionally confirmed in pigs and humans in the Philippines [3], [4]. Although no human case of filovirus hemorrhagic fever has been reported in Asian countries, the discovery of REBOV suggests the existence of REBOV in some wild animal species in Asia.

It is suspected that filoviruses persist in some species of fruit bats that may serve as natural reservoirs [5]–[7]. However, it is still unknown whether these bats continuously maintain filoviruses and act as a potential source of virus transmission to humans. Epidemiological studies suggest that index cases in outbreaks have often been linked to direct contact with apes presumably infected through bats or another reservoir species in Africa [8]. Information on the reservoirs and potential amplifying hosts for filoviruses in Asia is lacking. In this study, we focused on orangutans in Indonesia in the same geographic region as the Philippines. Until now, filovirus infection has never been reported in any animal species, including humans and nonhuman primates, in Indonesia. However, considering the geographical position of the Indonesian islands that provide habitats for various wild animals similar to those in the Philippines [9], which is the only EBOV-affected country so far reported in Asia, Indonesia may be at risk for filovirus infection.

We previously established a highly sensitive enzyme-linked immunosorbent assay (ELISA) with recombinant viral glycoprotein (GP) antigens derived from six different species of filoviruses (ZEBOV, SEBOV, CIEBOV, BEBOV, REBOV, and MARV) [10]. Since the antibody response to GP is likely filovirus species-specific due to the larger genetic variability with this protein, this assay would be helpful for retrospective seroepidemiologic surveys aimed at detecting species-specific antibody responses. By using this ELISA, we screened 353 orangutan serum samples for filovirus-specific IgG antibodies.

## Results

Orangutan serum samples were screened for IgG antibodies specific to each species of filoviruses (Figures S1 and S2), and all optical density (OD) values obtained by ELISA were analyzed statistically. Based on the distribution of the samples (Figure S3), we reasonably assumed that the big peak represented the negative sample population, and the outliers (*P*<0.01) with significantly higher OD values did not belong to the negative group. Thus, these statistical outlier samples were considered positive.

**Figure 1:**
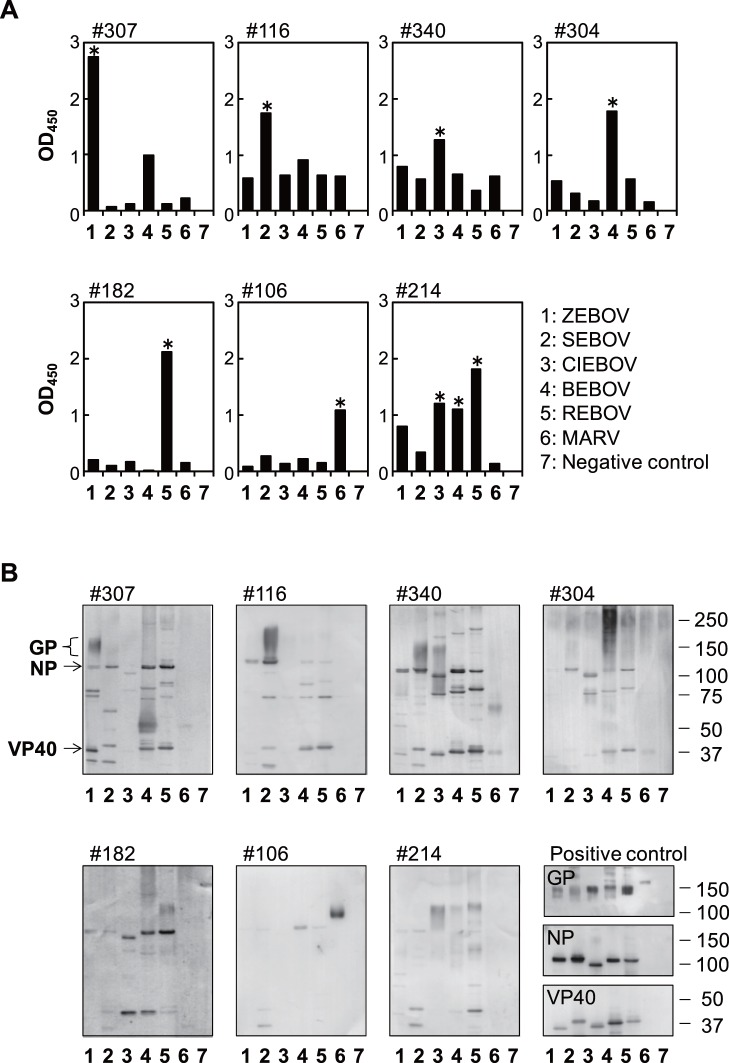
Filovirus species-specificity of IgG antibodies detected in orangutan sera. Serum samples diluted 1∶100 were tested for IgG antibodies reacting with soluble GP antigens in ELISA (A) and with GP, NP, and VP40 in Western blotting (B). Representative data for ZEBOV (#307), SEBOV (#116), CIEBOV (#340), BEBOV (#304), REBOV (#182), MARV (#106), and multiple (#214) GP-positive samples are shown. Asterisks indicate significantly higher OD values determined by the Smirnov-Grubbs rejection test (*P* < 0.01). VLPs consisting of GP, NP, and VP40 were used for Western blotting, and rabbit and mouse antisera or mouse monoclonal antibodies ZGP43/3.7 and AGP127-8 was used as positive controls as described in Materials and Methods. 1, ZEBOV; 2, SEBOV; 3, CIEBOV; 4, BEBOV; 5, REBOV; 6, MARV; 7, negative control.

Positive samples were then analyzed for species-specificity among filoviruses by comparing OD values given by each GP antigen. Representative data are shown in Figure 1. We found that some of the samples positive for one EBOV species were also positive for other EBOV species (e.g., #214 in Figure 1A), indicating that anti-EBOV IgG antibodies had cross-reactivity to some extent among species as demonstrated by previous studies [10], [11]. Unexpectedly, the majority of the positive samples showed strong specificity for ZEBOV, SEBOV, CIEBOV, or BEBOV (e.g., #307, #116, #340, or #304, respectively, in Figure 1A), all of which have thus far been found only in Africa. It was noted that none of these EBOV-positive samples were significantly cross-reactive to the MARV antigen. Conversely, MARV-positive samples showed strict specificity to the MARV antigen only (e.g., #106 in Figure 1A), which was consistent with a previous study [10]. Endpoint antibody titers of positive samples ranged between 1∶100 and 1∶25600 (Table 1). Since these positive samples exhibited distinct specificity either to the EBOV or MARV GP antigen, it was confirmed that the high OD values given by these samples were not due to nonspecific antibody reaction to the bovine serum albumin used for blocking or impurities contained in GP antigen preparations. In total, 1.4% (5/353) of the serum samples had the highest specificity for REBOV, which has been recognized as an Asian filovirus, whereas ZEBOV-, SEBOV-, CIEBOV-, BEBOV-, and MARV-specific IgGs were predominantly detected in 9.3% (33/353), 4.0% (14/353), 1.1% (4/353), 2.6% (9/353), and 1.7% (6/353) of the sera, respectively (Table 2). By contrast, IgM antibodies specific to ZEBOV, SEBOV, CIEBOV, BEBOV, REBOV, and MARV were detected only in 0.8% (3/353), 1.4% (5/353), 1.1% (4/353), 0% (0/353), 0.8% (3/353), and 0.3% (1/353) of the sera, respectively (Figures S4 and S5). While ongoing infection might be suggested in these animals, its frequency seemed to be limited.

**Table 1:** Serum IgG antibody titers of the positive sera.

	ELISA endpoint titers^1^				
Species	100	400	1600	6400	25600
ZEBOV	1^2^	7	20	1	4
SEBOV	0	5	9	0	0
CIEBOV	1	1	2	0	0
BEBOV	1	1	4	3	0
REBOV	0	0	4	1	0
MARV	1	2	3	0	0

1 Titers were expressed as the reciprocal of the highest dilution which gave an OD value above background.

2 Number of the samples with indicated titers are shown.

**Table 2:** Filovirus species specificity of the serum IgG antibodies detected in orangutans in East and Central Kalimantan.

	Positive rates (number positive/total)^1^					
Area	ZEBOV	SEBOV	CIEBOV	BEBOV	REBOV	MARV
East Kalimantan	5.3% (10/190)^2^	5.8% (11/190)	1.1% (2/190)	3.7% (7/190)	1.6% (3/190)	2.6% (5/190)
Central Kalimantan	14.1% (23/163)^2^	1.8% (3/163)	1.2% (2/163)	1.2% (2/163)	1.2% (2/163)	0.6% (1/165)
Total	9.3% (33/353)	4.0% (14/353)	1.1% (4/353)	2.6% (9/353)	1.4% (5/353)	1.7% (6/353)

1 The filovirus species for which each EBOV-positive sample had the highest OD value in the GP-based ELISA was selected when a sample showed cross-reactivity to GPs of multiple species.

2 A significant difference was found in ZEBOV positivity between East and Central Kalimantan (*P* = 0.0037).

To further confirm the IgG reactivity to filovirus proteins, GP-ELISA-positive samples were examined by immunoblotting using virus-like particles (VLPs) [10], [12], [13] consisting of GP, viral nucleoprotein (NP) and matrix protein (VP40) as target antigens (Figure 1B). We found that IgG in EBOV GP-positive samples bound to GP and also to approximately 90–104 kD and 35–40 kD proteins representing EBOV NP and VP40, respectively [1], and cross-reactivity to multiple EBOV species was appreciable for NP and VP40 but not significantly for GP in most of the samples tested. Overall, reactivity to NP and VP40 was observed in 78.5% (51/65) and 66.2% (43/65) of the EBOV GP-positive samples, respectively. Taken together, these results strongly suggested the presence of filovirus-specific IgG antibodies in these orangutan sera.

In summary, 18.4% (65/353) and 1.7% (6/353) of the samples were found to be seropositive (IgG) for EBOV and MARV GP antigens, respectively (Table 3) and detected anti-EBOV IgG antibodies showed specificity to various EBOV species, including African ZEBOV, SEBOV, CIEBOV, and BEBOV (Table 2). Ten (#110, #127, #164, #166, #214, #220, #319, #321, #378, and #382) out of 65 EBOV IgG positive samples showed cross-reactivity to multiple EBOV species (Figures S1 and S2). No significant difference was found in the overall positivity between genders or sampling locations, though there might be a geographical difference in virus species between East and Central Kalimantan (Table 2).

**Table 3:** Seroprevalence of filoviruses in orangutans in East and Central Kalimantan.

Area	Positive rates (number positive/total)					
	Ebola virus^1^			Marburg virus		
	Male	Female	Total	Male	Female	Total
East Kalimantan	14.9% (17/114)	21.1% (16/76)	17.4% (33/190)	2.6% (3/114)	2.6% (2/76)	2.6% (5/190)
Central Kalimantan	19.5% (17/87)	19.7% (15/76)	19.6% (32/163)	1.1% (1/87)	0% (0/76)	0.6% (1/163)
Total	16.9% (34/201)	20.4% (31/152)	18.4% (65/353)	2.0% (4/201)	1.3% (2/152)	1.7% (6/353)

1 Numbers of samples positive for any of the EBOV species were counted. There was no significant difference in positivity between males and females or between East and Central Kalimantan.

## Discussion

In this study, we detected filovirus GP-specific IgG antibodies in the orangutan serum samples collected in Indonesia, whereas previous seroepidemiological studies of primates mainly detected antibodies to NP and VP40 antigens in ELISA and/or immunofluorescent tests. As compared with NP or VP40, GP is, in general, considered to induce antibodies more specific or little cross-reactive among filoviruses and other pathogens, thus enabling us to detect filovirus species-specific antibodies. There might be a possibility that our results were due to the cross-reactivity to related known viruses (e.g., paramyxoviruses and rhabdoviruses) or carbohydrates on the GP molecules. However, since the majority of the positive samples showed significant specificities to GPs of particular filovirus species, it seems unreasonable to assume that all filovirus species have unique counterpart pathogens or sugar chains independently causing nonspecific antibody reactions to the respective filovirus species.

In ELISA, we were not able to use the cutoff OD value based on control orangutan populations. Instead, we first considered the use of positive or negative control samples from other nonhuman primates used in experimental infections. However, since experimental infection of nonhuman primates with EBOV (particularly ZEBOV) is highly lethal, positive control sera from naturally recovered animals were not available. In addition, the distribution of OD values obtained from small numbers of uninfected laboratory animals did not work as a good negative control for comparison with samples collected from wild animals since samples from laboratory animals generally showed lower average OD values with smaller deviations than those from wild animals. Differences in the reactivity of the peroxidase-conjugated secondary antibody among primate species (e.g., orangutans vs. macaques) should also be a potential problem. Therefore, to determine the statistical significance of each OD value (i.e., to assume as positive), we employed the Smirnov-Grubbs rejection test which detects outliers. However, since this approach is simply based on statistical constructs, there might be some other reasons for the outliers, depending on the population size and considerable individual variability of wild animals. To verify our ELISA data, it would be helpful to perform other serological assays such as neutralization tests.

This study suggests the existence of multiple species of filoviruses or unknown filovirus-related viruses in Indonesia, some of which are serologically similar to African EBOVs, and also hypothesizes that filoviruses or filovirus-related viruses might be more widely distributed in Asian countries than assumed hitherto. Isolation of the virus will be needed to confirm these results and to understand the ecology of filoviruses in Asia, particularly to answer following questions; (i) Is there any known filovirus species other than REBOV in Asia? (ii) Are Asian filoviruses comparatively low pathogenic and asymptomatically maintained in the nonhuman primate populations? (iii) Are the viruses introduced into primates from putative natural reservoir animals? (iv) Is there a risk of virus transmission to humans through contact with nonhuman primates and/or yet unidentified natural reservoir hosts in Asia?

Hypothetically, filoviruses persist in endemic areas in wild animal species that are resistant or tolerant to the virus and serve as natural reservoirs. Epidemiological information on several EBOV and MARV outbreaks suggests that some species of bats may be natural reservoir animals for filoviruses [5]. Indeed, multiple infectious MARV strains were recently isolated from Egyptian fruit bats *(Rousettus aegyptiacus*) [6]. On the other hand, EBOV has never been isolated from any bat species, although fruit bats (*Hypsignathus monstrosus, Epomops franqueti,* and *Myonycteris torquata*) captured in the outbreak areas in Gabon and the Democratic Republic of Congo were found to have small amounts of ZEBOV genomic RNA and virus-specific antibodies [7]. EBOV-specific antibodies were also detected in the fruit bat species *Rousettus aegyptiacus* and *Rousettus amplexicaudatus* captured in Africa and the Philippines, respectively [14], [15], suggesting that these bat species are potential natural reservoirs for EBOV. Indonesia and other Asian countries provide a large habitat for fruit bats, including the Rousettus bats [9], [16]. Thus, it should be clarified whether fruit bats may act as a potential source of filovirus transmission to nonhuman primates and whether these bats continuously maintain EBOV or MARV.

Apes are highly susceptible to filovirus infection and exhibit lethal disease similar to that in humans, so these species have not been considered to be reservoir animals [8], [17]. Indeed, populations of gorillas and chimpanzees have declined markedly as a result of ZEBOV infection in central Africa [18]. However, the relatively high seroprevalence of multiple filovirus species in Indonesian orangutans may suggest asymptomatic or at least nonlethal infection due to their resistance to filoviruses. Indeed, experimental infection of nonhuman primates with ZEBOV, SEBOV, and REBOV showed that the lethality of the viruses seemed to vary depending on the primate species [19], [20]. Alternatively, it can also be hypothesized that there are some unidentified filoviruses and/or filovirus-related viruses that potentially belong to the filovirus family but are not highly virulent to primates. This hypothesis may explain the relatively high prevalence of IgG antibodies and low mortality in orangutans. Such viruses may have life cycles distinct from those of highly virulent viruses and could be co-evolved with nonhuman primates. In this hypothesis, it is speculated that filoviruses that are lethal for primates may be maintained in some other animal species or emerge from nonlethal filoviruses persisting in wild primates and/or some other wild animals through mutations. It should be noted that filovirus pathogenicity might be altered with only a few amino acid substitutions during rapid adaptive selection in particular animals, as demonstrated in the process for producing rodent models of EBOV infection [21], [22].

Because the population density and numbers of orangutans in Indonesia do not seem to be high enough to maintain any virus that causes an acute infection, it seems unlikely that they could serve as reservoir hosts unless filoviruses are somehow able to cause chronic infection in orangutans, as is the case with a persistent infection model of measles virus, other member of the *Mononegavirales* [23]. Instead, they were presumably infected through direct or indirect contact with another animal species that might be the reservoir and/or amplifying hosts of filoviruses. However, we assume that some species of wild primates could be infected without significant illness and able to serve as carrier or amplifying hosts of filoviruses in some circumstances, as is suggested for other viral zoonotic diseases such as sylvatic yellow fever, chikungunya, and Kyasanur forest disease [24], [25], [26]. Whereas these are vector-borne diseases, possible roles of arthropods in the ecology of filoviruses have also been speculated [27].

A point to note is the high likelihood of an EBOV and MARV source, including as yet unknown species, in nature. It is possible that the filoviruses include diverse members with different pathogenicity and different perpetuation mechanisms. Indeed, a new filovirus, named Lloviu virus, was recently detected from long-fingered bats (*Miniopterus schreibersii*) in Spain [28]. This insectivorous bat species is widely distributed in Oceania, southern Europe, southern Africa, and southeast Asia [29]. Our findings emphasize the need for a joint risk assessment regarding filovirus infection in Asia at the interface between environment, domestic animals and human populations. Further laboratory and ecological investigations are needed to understand how fruit bats and nonhuman primates may play roles in maintaining filoviruses and potentially introducing them into humans.

## Materials and Methods

### Animals and Sera

Three hundred fifty-three serum samples (from 201 males and 152 females) were collected from wild-caught healthy orangutans (*Pongo pygmaeus*) in East (Kutai Kartanegara) and Central (Palangka Raya) Kalimantan provinces in Indonesia from December 2005 to December 2006 (Table S1). All samples were collected originally for serological diagnosis of influenza and/or mycobacteriosis and tested in the ABSL-3 facility belonging to Airlangga University, Surabaya, Indonesia. The Indonesian government, for conservation strategies, conducts a regular monitoring of infectious diseases in orangutan populations. Under the direction of the Ministry of Forestry, Indonesia, orangutans were carefully captured by at least 4 people using nets to investigate their health conditions. All captured orangutans were registered to give their names. Animals were anesthetized by intramuscular injection of Ketamine and Xylaxin, and blood samples were taken from the brachial vein. After taking the samples, each animal were kept in a single cage. After quarantine (e.g., tuberculosis, hepatitis B, and so on), they were released to the forest if they showed negative results for the diseases and normal social behavior. All the captured orangutans released to the forest were monitored at least around six months. Part of the previous collection was used for this study. Animal works were performed under the approval of Airlangga University Ethical Committee.

### ELISA

Filovirus GP-based ELISA was performed as described previously [10]. Briefly, His-tagged soluble recombinant GPs of ZEBOV (strain Mayinga), SEBOV (strain Boniface), CIEBOV (strain Cote d’Ivoire), BEBOV (strain Bundibugyo), REBOV (strain Pennsylvania), and MARV (strain Angola) were purified from the supernatants of cultured 293T human embryonic kidney cells [30], [31] transfected with pCAGGS expressing each GP by using the Ni-NTA Purification System (Invitrogen). ELISA plates (Nunc Maxisorp) were coated with the GP antigens (100 ng of GP/50 µl/well) or negative control antigens (FCS-derived proteins non-specifically bound to Ni-beads) [10], followed by blocking with 3% skim milk (150 µl/well). Orangutan serum samples diluted appropriately (1∶100 or serially 4-fold from 1∶100) were added and incubated for 1 hour at room temperature. The bound antibodies were visualized with peroxidase-conjugated goat anti-monkey IgG or IgM (ROCKLAND) and 3,3′,5,5′-tetramethylbenzidine (Sigma). Reaction was stopped by adding 1 N sulfuric acid and the optical density (OD) at 450 nm was measured. To offset the nonspecific antibody reaction, the OD values of each sample were subtracted from those given by the control antigen. Assays were duplicated and averages were used for further analysis.

### SDS-PAGE and Western Blotting

To generate VLPs, 293T cells were transfected with plasmids expressing the major viral structural proteins, GP, NP, and VP40, of ZEBOV, SEBOV, CIEBOV, BEBOV, REBOV, and MARV. After 48 hours, supernatants were overlaid on 25% sucrose and ultracentrifuged at 28,000 g at 4°C for 1.5 hours, and VLPs were recovered from the pellet. The protein amounts in the VLPs were quantified by Western blotting using MAb ZGP42/3.7 [32] or AGP127-8 [33], as described previously [10]. Supernatants from 293T cells transfected with an empty vector, pCAGGS, were used as a negative control. VLP lysates were electrophoresed by SDS-PAGE on 5–20% SuperSep (Wako), and blotted on PVDF membranes (Millipore). The membranes were incubated with the serum samples (1∶100 dilution) or control antibodies, followed by incubation with peroxidase-conjugated goat anti-monkey IgG (ROCKLAND). The bound antibodies were visualized with Konica immunostaining HRP-1000 (Konica). Anti-EBOV NP rabbit antisera were produced by immunization with keyhole limpet hemocyanin-conjugated synthetic peptides corresponding to amino acid positions 542–555 (CAPLTDNDRRNEPSG), 631–644 (CQGSESEALPINSKK), 635–652 (NQVSGSENTDNKPH), 628–641 (QSNQTNNEDNVRNN), and 630–643 (TSQLNEDPDIGQSKC) of ZEBOV, SEBOV, CIEBOV, BEBOV, and REBOV NPs, respectively. For the anti-EBOV VP40 mouse antiserum, the synthetic peptide corresponding to amino acid positions 86–99 (KVLMKQIPIWLPLG) of EBOV VP40 were used. Mouse monoclonal antibody ZGP42/3.7 and AGP127-8 was used to detect EBOV and MARV GPs, respectively.

### Statistics

Since there were no control orangutan samples either positive or negative for filovirus antibodies, we were not able to set the cutoff value of OD based on such control populations. Instead, to determine statistical significance of each OD value, we applied the Smirnov-Grubbs rejection test, which is widely used to detect significantly higher or lower values (i.e., outliers) that do not belong to the population consisting of all other values in the data set. Based on this distribution of the samples (Figure S3), we detected statistical outliers.

Briefly, the highest OD value was first picked up, and the T value (T_OD highest_  =  |OD_highest_ – OD_Average1-2118_|/OD_Standard deviation1-2118_) was calculated for its statistical significance based on the critical values given by the Smirnov-Grubbs test (*n* = 2118; T = 4.4218; *P*<0.01). If it was considered to be an outlier, the T value for the second highest OD value was then similarly tested without the highest one (T_OD 2nd highest_  =  |OD_2nd highest_ – OD_Average1-2117_|/OD_Standard deviation1-2117_) (*n* = 2117; T = 4.4217; *P*<0.01). These steps were repeated until the T value fell to below the level of statistical significance (*P* < 0.01), and detected outlier samples were regarded as positive (i.e., samples that did not belong to the population formed by the rest of samples with lower OD values). Differences in seroprevalence between genders and sampling locations were analyzed using Fisher’s exact test.

## Supporting Information

Figure S1 **IgG antibodies detected in the sera collected in East Kalimantan.** Serum samples were tested (1∶100 dilution) for IgG antibodies reacting with soluble GP antigens derived from ZEBOV, SEBOV, CIEBOV, BEBOV, REBOV, and MARV in ELISA as described in Materials and Methods. Asterisks indicate significantly higher OD values determined by the Smirnov-Grubbs rejection test (*P* < 0.01).
(TIF)

Figure S2 **IgG antibodies detected in the sera collected in Central Kalimantan.** The experimental conditions and statistics were the same as those described in Figure S1. Seven samples (ID# 364, 406, 418, 423, 436, 451 and 457) are absent.
(TIF)

Figure S3 **The frequency distribution of the orangutan serum samples according to OD values obtained by ELISA.** All OD values obtained in filovirus GP-based ELISA for 5 EBOV and 1 MARV species were analyzed concurrently (n =  118). The frequency distribution chart reveals that the sample population consists of a major single peak with low OD values (approximately ∼0.8) and outliers (*P* < 0.01) with high OD values (approximately 1.0∼). The statistical significance of each OD value obtained by ELISA was evaluated by using the Smirnov-Grubbs rejection test, which is widely used to detect significantly higher and lower values (i.e., outliers).
(EPS)

Figure S4 **IgM antibodies detected in the sera collected in East Kalimantan.** Serum samples were tested (1∶100 dilution) for IgM antibodies reacting with soluble GP antigens derived from ZEBOV, SEBOV, CIEBOV, BEBOV, REBOV, and MARV in ELISA as described in Materials and Methods. Asterisks indicate significantly higher OD values determined by the Smirnov-Grubbs rejection test (*P* < 0.01).
(TIFF)

Figure S5 **IgM antibodies detected in the sera collected in Central Kalimantan.** The experimental conditions and statistics were the same as those described in Figure S4. Seven samples (ID# 364, 406, 418, 423, 436, 451 and 457) are absent.
(TIFF)

Table S1 Summary of the orangutan serum samples analyzed.
(DOCX)
