# Expression of Concern: Serological Evidence of Ebola Virus Infection in Indonesian Orangutans

**DOI:** 10.1371/journal.pone.0060289

**Published:** 2013-03-20

**Authors:** The PLOS ONE Editors

Following the publication of this article, the editors were alerted to concerns over the accuracy and clarity of information reported regarding the source of the samples included in the study.

The editors were contacted by representatives of the Borneo Orangutan Society who indicated that the samples included in this study were collected by the society for surveillance purposes as per the requirements under Indonesian law, and submitted to Airlangga University for routine tuberculosis tests. The Borneo Orangutan Society indicated that the samples originate from animals kept in rehabilitation camps and that they had no knowledge of the additional tests carried out for the purposes of this research.

We have followed up with the authors in relation to these concerns and while we have received confirmation that the samples were collected for surveillance purposes, we have not received a satisfactory response to the request for specific details for the origin of each of the samples included in the analyses or the methodological steps involved in their collection.

In the light of this, the editors are issuing this Expression of Concern to make readers aware of the concerns over the origin as well as the methodology followed for the collection of the samples tested in the study. The editors have also raised the concerns in relation to this matter to the attention of representatives of Airlangga University and the Indonesian government.

