# Supplementary figures and images for: Serological Evidence of Ebola Virus Infection in Indonesian Orangutans
Source: PLoS One. 2012 Jul 18;7(7):e40740. doi: 10.1371/journal.pone.0040740 (PMC3399888; doi:10.1371/journal.pone.0040740)

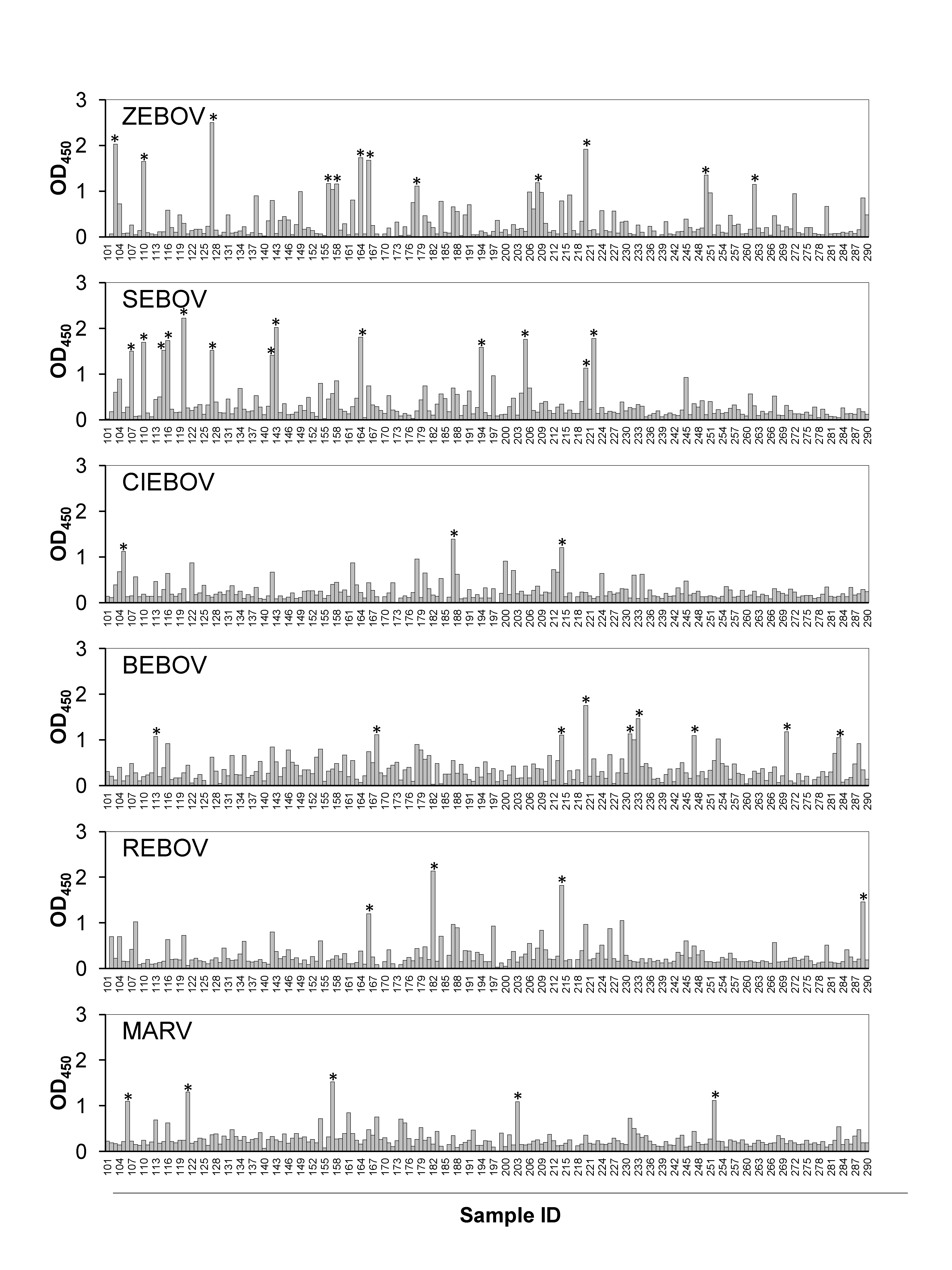

Supplement: Figure S1 — IgG antibodies detected in the sera collected in East Kalimantan. Serum samples were tested (1∶100 dilution) for IgG antibodies reacting with soluble GP antigens derived from ZEBOV, SEBOV, CIEBOV, BEBOV, REBOV, and MARV in ELISA as described in Materials and Methods. Asterisks indicate significantly higher OD values determined by the Smirnov-Grubbs rejection test (P < 0.01). (TIF) [file pone.0040740.s001.tif]

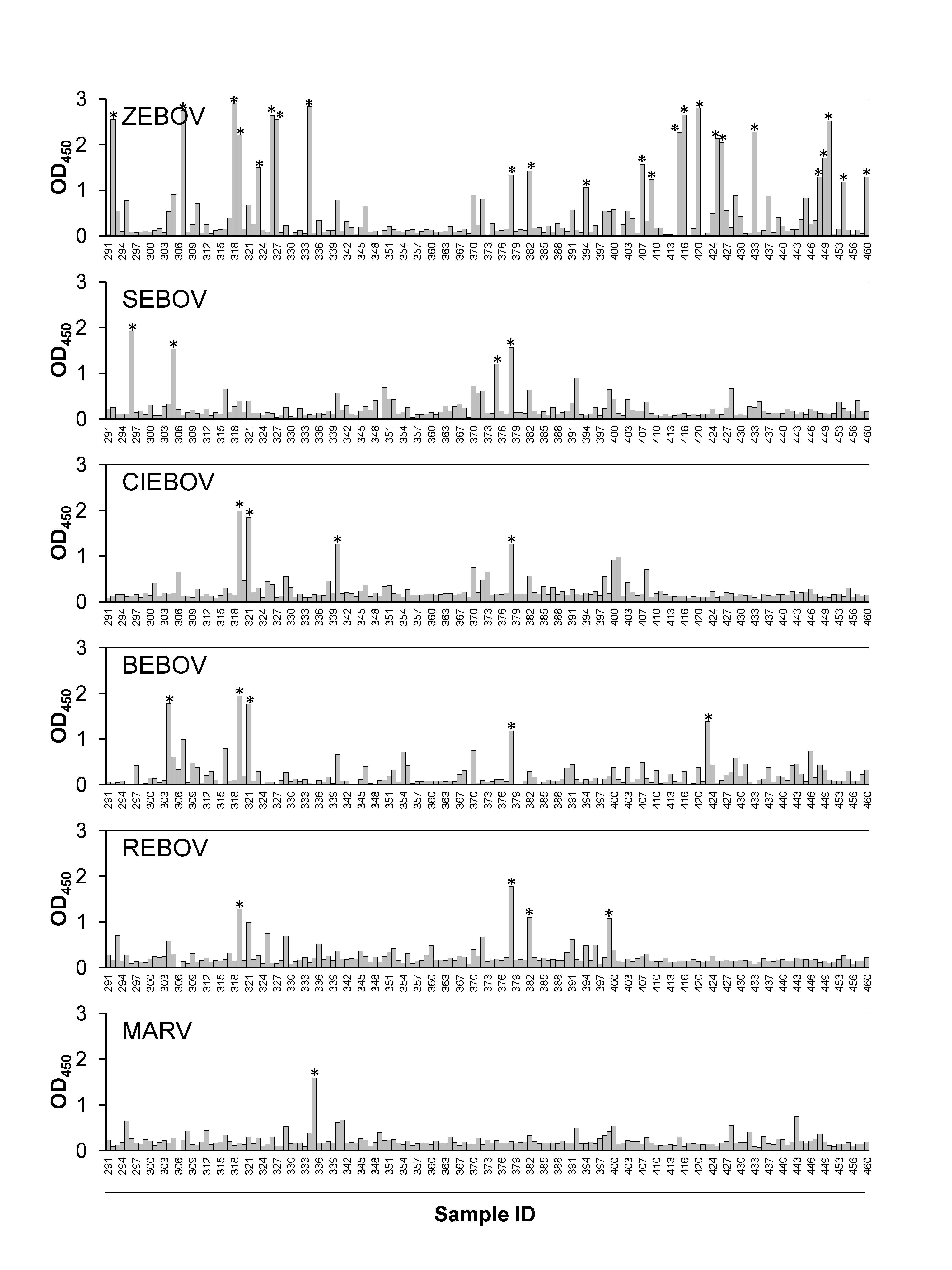

Supplement: Figure S2 — IgG antibodies detected in the sera collected in Central Kalimantan. The experimental conditions and statistics were the same as those described in Figure S1. Seven samples (ID# 364, 406, 418, 423, 436, 451 and 457) are absent. (TIF) [file pone.0040740.s002.tif]

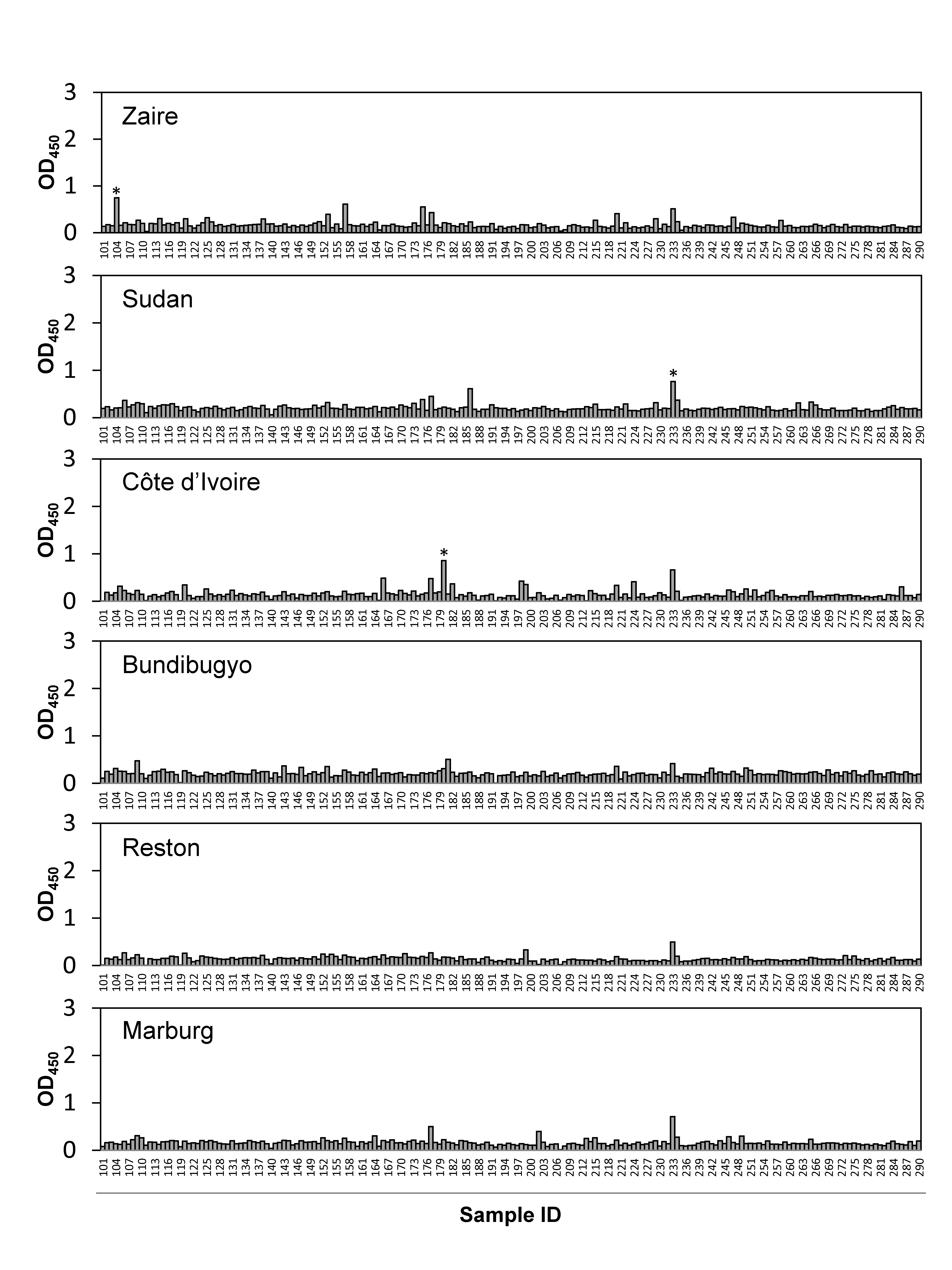

Supplement: Figure S4 — IgM antibodies detected in the sera collected in East Kalimantan. Serum samples were tested (1∶100 dilution) for IgM antibodies reacting with soluble GP antigens derived from ZEBOV, SEBOV, CIEBOV, BEBOV, REBOV, and MARV in ELISA as described in Materials and Methods. Asterisks indicate significantly higher OD values determined by the Smirnov-Grubbs rejection test (P < 0.01). (TIFF) [file pone.0040740.s004.tiff]

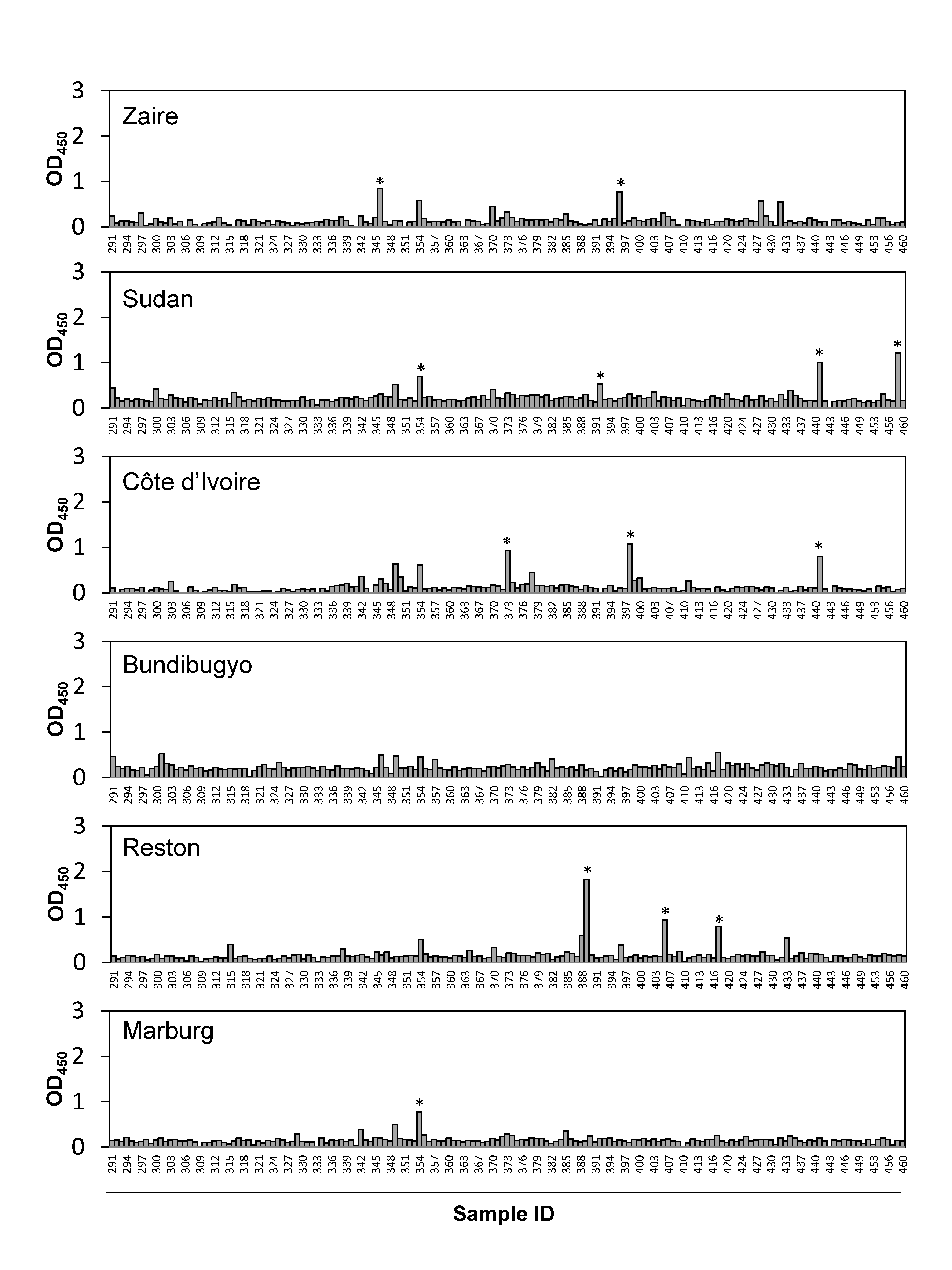

Supplement: Figure S5 — IgM antibodies detected in the sera collected in Central Kalimantan. The experimental conditions and statistics were the same as those described in Figure S4. Seven samples (ID# 364, 406, 418, 423, 436, 451 and 457) are absent. (TIFF) [file pone.0040740.s005.tiff]
