# Supplementary material for: Serological Evidence of Ebola Virus Infection in Indonesian Orangutans
Source: PLoS One. 2012 Jul 18;7(7):e40740. doi: 10.1371/journal.pone.0040740 (PMC3399888; doi:10.1371/journal.pone.0040740)
Supplement: Table S1 — Summary of the orangutan serum samples analyzed. (DOCX) [file pone.0040740.s006.docx]

**Table S1.** Summary of the orangutan serum samples analyzed

|  |  | Number of animals | |
| --- | --- | --- | --- |
| Area | Date^1^ | Male | Female |
| East Kalimantan | December 1, 2005 | 3 | 2 |
| East Kalimantan | January 1, 2006 | 1 | 1 |
| East Kalimantan | January 14, 2006 | 1 | 1 |
| East Kalimantan | January 28, 2006 | 1 | 2 |
| East Kalimantan | February 1, 2006 | 5 | 3 |
| East Kalimantan | February 3, 2006 | 2 | 2 |
| East Kalimantan | February 21, 2006 | 3 | 4 |
| East Kalimantan | February 23, 2006 | 4 | 4 |
| East Kalimantan | February 27, 2006 | 8 | 3 |
| East Kalimantan | March 10, 2006 | 7 | 4 |
| East Kalimantan | March 14, 2006 | 7 | 8 |
| East Kalimantan | March 16, 2006 | 14 | 6 |
| East Kalimantan | March 18, 2006 | 12 | 5 |
| East Kalimantan | March 19, 2006 | 2 | 4 |
| East Kalimantan | March 21, 2006 | 3 | 8 |
| East Kalimantan | March 28, 2006 | 20 | 12 |
| East Kalimantan | March 31, 2006 | 11 | 1 |
| East Kalimantan | April 1, 2006 | 8 | 2 |
| East Kalimantan | April 3, 2006 | 2 | 4 |
| Central Kalimantan | March 21, 2006 | 7 | 3 |
| Central Kalimantan | April 2, 2006 | 3 | 2 |
| Central Kalimantan | April 5, 2006 | 9 | 2 |
| Central Kalimantan | April 13, 2006 | 9 | 3 |
| Central Kalimantan | May 15, 2006 | 8 | 0 |
| Central Kalimantan | May 29, 2006 | 2 | 9 |
| Central Kalimantan | June 9, 2006 | 1 | 4 |
| Central Kalimantan | June 11, 2006 | 7 | 6 |
| Central Kalimantan | June 14, 2006 | 2 | 3 |
| Central Kalimantan | June 18, 2006 | 3 | 5 |
| Central Kalimantan | June 21, 2006 | 2 | 8 |
| Central Kalimantan | June 27, 2006 | 6 | 6 |
| Central Kalimantan | July 8, 2006 | 5 | 4 |
| Central Kalimantan | July 19, 2006 | 1 | 3 |
| Central Kalimantan | August 6, 2006 | 2 | 5 |
| Central Kalimantan | August 20, 2006 | 6 | 4 |
| Central Kalimantan | August 23, 2006 | 3 | 1 |
| Central Kalimantan | September 5, 2006 | 1 | 3 |
| Central Kalimantan | September 10, 2006 | 1 | 4 |
| Central Kalimantan | November 2, 2006 | 2 | 1 |
| Central Kalimantan | November 14, 2006 | 2 | 0 |
| Central Kalimantan | November 24, 2006 | 2 | 0 |
| Central Kalimantan | December 1, 2006 | 1 | 0 |
| Central Kalimantan | December 7, 2006 | 2 | 0 |
|  | Total | 201 | 152 |

^1^Serum samples were collected from different individuals on indicated dates.
